# Auditory brainstem response in gas station attendants

**DOI:** 10.5935/1808-8694.20120035

**Published:** 2015-10-20

**Authors:** Lenita da Silva Quevedo, Tania Tochetto, Marcia Amaral Siqueira, Márcia Salgado Machado

**Affiliations:** a MsC in Human Communicaton Disorders – Federal University of Santa Maria; Temporary Substtute Professor of Speech and Hearing Therapy – University of Passo Fundo; b PhD in Human Communicaton Disorders – Federal University of São Paulo; Associate Professor of Speech and Hearing Therapy of the Federal University of Santa Maria; c MsC in Human Communicaton Disorders – Federal University of Santa Maria; Speech and Hearing Therapist - Centro de Referência em Saúde do Trabalhador de Santa Maria; d MsC in Human Communicaton Disorders – Federal University of Santa Maria; Assistant Professor at the Department of Speech and Hearing Therapy of the Federal University of Health Sciences of Porto Alegre; Universidade Federal de Santa Maria

**Keywords:** evoked potentials, auditory, brain stem, hearing, solvents

## Abstract

Ototoxicity of organic solvents can affect the hearing system up to the cochlea level and the central structures of hearing.

**Objective:**

To evaluate the neurophysiological integrity of the hearing system in subjects exposed to fuels using ABR.

**Method:**

Prospective study. We evaluated attendants from three gas stations in Santa Maria/RS. The sample had 21 subjects, who were evaluated by auditory brainstem response.

**Results:**

We found an alteration in the absolute latencies of Waves I and III and in all the interpeak latencies, in the right ear. In the left ear there was a change in the absolute latencies of all Waves, and in all the interpeak intervals. A change in the interaural difference of Wave V was found in 19% of the individuals. In the group exposed for more than five years, there were subjects with a statistically significant changes: in the I-V interpeak of the right ear; in the absolute latency of Wave I and in the III-V interpeak of the left year.

**Conclusion:**

Exposure to fuels can cause alterations in the central hearing system.

## INTRODUCTION

Literature has shown that the ototoxicity of organic solvents may reach the auditory system, not only at a cochlear level, but it may also affect the central structures of hearing.

The effects of some neurotoxic substances may appear only after repeated exposures along weeks or even years, such as for example, regular breathing the vapors from a solvent in the work place^1^.

Each body system may be affected in a different way by toxic substances; however, the nervous system is particularly vulnerable. One of the reasons for this vulnerability is that certain regions of the brain and the nerves are directly exposed to chemical substances in the blood. Moreover, contrary to other cells which make up the body, neurons usually do not regenerate, since the damage caused by toxic agents to the brain or spinal cord, is usually permanent^1^.

It has been noticed in rats and in human beings that the higher concentration of toluene happens in the brainstem^2^. This fact proves that organic solvents can easily cross the blood-brain barrier after inhalation, and produce CNS effects similar to those from alcohol and benzodiazepines^3^.

Thus, individuals exposed to chemical products are more prone to CNS changes, especially those exposed in their occupation, in which exposure is daily and continuous.

It is important to stress that the traditional way of investigating occupational hearing loss only by means of threshold tonal audiometry (TTA) may not be enough or adequate when one considers the effects of exposure to chemical agents^4^. The neurophysiological assessment is useful in the identification of the adverse effects of neurotoxic substances^1^. The Brainstem Auditory Evoked Potential (BAEP) acts as an aid in the detection of sensorineural hearing loss of workers who operate in noisy places and are exposed to neurotoxic substances, because these substances reach the brainstem auditory pathways and not necessarily the hair cells^5^.

The exact place and the mechanism of action of solvents are not fully understood. Most of the CNS is dedicated to the identification and processing of auditory information; nonetheless, it is still necessary to fully understand the effects of solvents on the central auditory nervous system (CANS)^6^. It is of paramount importance to understand how the CANS works, how it can be damaged and how to assess it^7^.

Therefore, the present investigation aimed at assessing the neurophysiological integrity of the auditory system all the way to the brainstem, by means of the Brainstem Auditory Evoked Potentials.

## METHOD

This study is of quantitative nature.

We investigated gas station attendants in the city of Santa Maria/RS, exposed to fuels.

Inclusion criteria for the gas stations were: gas stations in the city of Santa Maria/RS, open 24 hours and selling more gas than the others. According to these criteria, we selected three gas stations, gathering a total of 78 workers.

The sound pressure level was measured in the three gas stations investigated, aiming at excluding the possibility of hearing changes caused by high sound pressures. The measurement was carried out with a Q-400 dosimeter, adjusted to the compensation scale “A” and slow response velocity. The device was placed on the belt of the worker and a microphone was attached near his/her ear, without interfering in their movements. The dosimeter was installed at 8 am and removed at 4 pm - time span corresponding to their daily working hours.

The inclusion criteria for the subjects were: not having ear problems in the past, have normal auditory thresholds (hearing thresholds below 25 dB in all the frequencies assessed - 250 to 8000 Hz) and type A tympanometric curve; have less than 40 years of age and not having been exposed to noise, organic solvents or pesticides; and not under use of ototoxic medication.

Of the 78 individuals who work in the three gas stations, after fitting the inclusion criteria, there were 21 left, three females and 18 males.

All the individuals worked pumping gas, and were exposed to the vapors of the organic solvents present in gasoline. The time of exposure varied between one and fifteen years.

The individuals were tested after reading and signing the Informed Consent Form.

The procedures carried out were: inspection of the external acoustic canal, TTA, tympanometry, acoustic reflex testing and BAEP.

The external acoustic canal inspection was carried out using the *Klinic Welch*-*Allyn* clinical otoscope, aiming at looking for wax or any other change that could prevent the tests from being carried out or which could alter the results of such tests.

The audiological assessments were carried out in a soundproof booth. Threshold tonal audiometry was carried out with an AC40 audiometer from *Interacoustics*, with TDH-39 headphones. Tympanometry and the acoustic reflexes were tested through the AT 235 middle ear analyzer from *Interacoustics*.

Following the inclusion criteria check, the subjects were submitted to electrophysiological assessment by means of BAEP. We used the two-channel Eclypse EP 15 device from *Interacoustics*. The test was carried out in a silent environment, with the individual laying down on his/her back, without sedation or any other medication. After cleaning the skin with 70% alcohol solution and mild exfoliation with an abrasive paste, a conductive electrolytic paste was placed between the skin and the leads, and then they were fixed with micropore tape. In order to capture the electrical potentials we used non-disposable surface electrodes. The positioning of the electrodes corresponded to the definitions from the 10/20 international system, with the negative ones fixed to the mastoids (A1 and A2), left and right – respectively, the positive on the forehead, closer to the vertex (Cz) and the ground electrode was placed on the frontal region (Fpz). The electrodes impedances were checked, starting the acquisitions whenever values below 3 kOhms were found. The individuals were instructed to maintain their eyes closed during the acquisitions and avoid any body movement. The stimulus utilized was the monoaural click, at the intensity of 80 dB HL, introduced by a transducer for at least two times in each ear, in order to check for wave overlapping. The stimulus frequency spectrum was between 500 and 8000 Hz, with an individual duration of 100 microseconds and thin polarity. The results were seen in a 12ms window, and we used a low pass filter adjusted at 3000 Hz. Sensitivity varied between 10 and 40µV and the clicks were presented at the frequency of 39.1/s.

The absolute latency values of waves I, III and V; the I-III, III-V and I-V interpeak values, as well as wave V interaural difference were analyzed according to the standardization of the device.

As a normality standard, we considered the reference values in the equipment utilized, considering normal neural conduction bearers in the auditory pathway those individuals who had absolute latencies and interpeak latencies within the values established in the equipment – with the specific standard deviation value () for each ear. The normal values established in the equipment are: wave I absolute latency of 1.8 ms with SD of 0.40; wave III absolute latency of 3.8 ms with SD of 0.167; and wave V absolute latency of 5.767 ms with an SD of 0.60.

The absolute latencies and the interpeak intervals were also analyzed in relation to the duration of exposure of the subjects, according to suggestions from prior stu-dies^8^, ^9^. The exposure time ranges were set on: from one to three years, from three years and one month to five years and more than five years of exposure.

This research Project was approved by the Ethics in Research Committee in Human Beings of our institution, under protocol # 23081.011007/2010-80.

Waves I, III and V absolute latencies, as well as the interpeak interval, were analyzed on the right and left ears separately, because we found a statistically significant difference between the ears (test *t*).

The binominal test was used to analyze the absolute latencies and interpeak of the BAEP, as well as for the analysis of results in relation to the time of exposure. The level of statistical significance was set on 5% (*p*<0.05).

## RESULTS

The subjects investigated were: 14.3% females and 85.7% males. The mean age of the individuals was 29.76 years (SD = 5.2).

**Figure 1:**
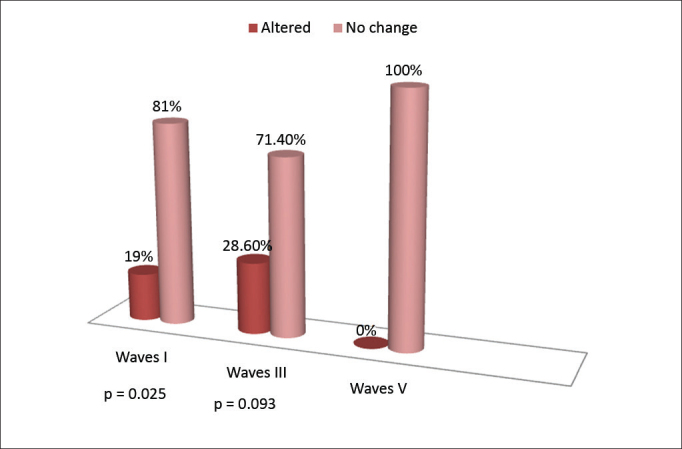
Alteration on the absolute latencies of waves I, III and V, on the right ear.

On the right ear we found changes in the absolute latencies of waves I and III; notwithstanding, the absolute latency of waves I and III, however, the wave V absolute latency was normal in all the individuals assessed. However, without statistically significant differences (*p* > 0.05), wave III had the highest number of subjects with latency changes (Figure 1).

We observed changes in all the latencies of the interpeak intervals on the right ear: I-III (38.1% of the individuals), III-V (4.8% of the individuals) and I-V (14.3% of the individuals), where the I-III interpeak interval had the highest number of individuals with changes, without however, a statistically significant difference (*p* > 0.05) between the number of patients with change and those without change in this interval.

**Figure 2:**
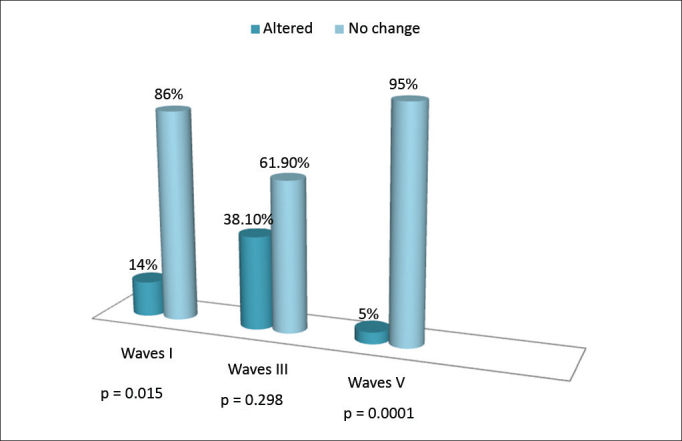
Alterations on the absolute latencies of waves I, III and V, on the left ear.

On the left ear we found changes in the absolute latencies of all the waves. As it happened to the right ear, the number of individuals with changed latency was higher in wave III (Figure 2).

Although the unaltered results were higher than the altered ones, we noticed changes in all the interpeak latencies of the left ear, where the I-III interpeak intervals (14.3%) and III-V (14.3%) had the highest number of individuals with changes, vis-à -vis the I-V interval (9.5%). However, there was no statistically significant difference in any of the intervals (*p* > 0.05).

**Figure 3:**
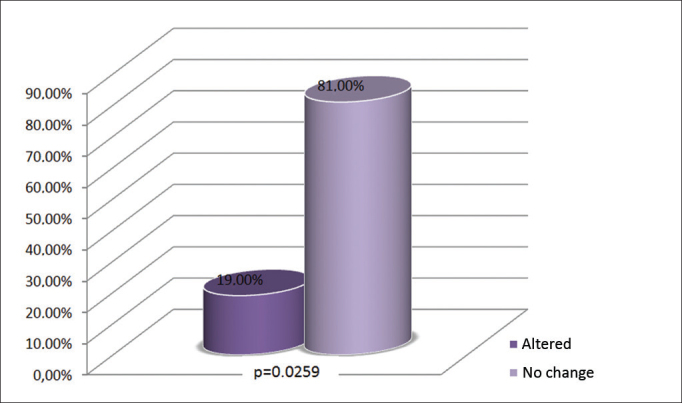
Alterations on the interaural difference of wave V.

Part of the subjects assessed had changes in the wave V interaural difference (19%), nonetheless, this value did not have statistical significance (Figure 3).

The interpeak and absolute latencies were also analyzed according to the time of exposure of the individuals, which was divided according to the literature^8^, ^9^.

Among the attendants exposed for at least three years, we found changes in all the individuals (n = 8), in the absolute latencies of waves I and V of the right ear; notwithstanding, this finding was not statistically significant. In addition, there was a statistically significant difference in the III-V interpeak interval change of the right ear (*p* = 0.0257) and on the absolute latency of wave V on the left ear (*p* = 0.0257).

In the group exposed between three years and one month to five years, changes to the right ear were seen in all the subject, namely: absolute latencies of waves I and V and all the interpeak intervals. On the left ear, the absolute latencies of waves I and V, the III-V interpeak intervals, as well as the interaural difference of wave V, were also changed in all the subjects (n=3). We did not find statistically significant change to the values of absolute and interpeak latencies.

In the group exposed for more than five years, the number of individuals with changes in the I-V interpeak interval on the right ear (*p* = 0.0173), on the absolute latency of wave I and in the III-V interpeak interval on the left ear (*p* = 0.0173). Moreover, all the subjects (n = 10) had changes on the wave V absolute latency, in both ears, as well as in the III-V (right ear) and I-V (left ear) interpeak intervals.

The measurement of sound pressure levels in each one of the gas stations showed that none of them had sound pressure levels above the tolerance limits established on the Attachment # 1 of NR-15 Standard. Therefore, we suppose that the changed results seen in the individuals of this study stem from the exposure to the chemical products which are components of the fuels.

## DISCUSSION

The absolute latencies of waves I and III on the right ear were altered on 19% and 20.60% of the individuals, respectively. On wave V we did not find changes. On the left ear there was an increase on the absolute latencies of the three waves (Figure 1). Wave III was the one with the highest number of individuals with changes, in the right ear (20.60%) as well as in the left ear (38.10%) (Figure 2). These results, although not statistically significant (*p* > 0.05), suggest changes to the distal portion of the auditory nerve and cochlear nucleus. Thus, as in this study, other authors^10^ also found greater changes to waves I and III, notwithstanding, wave I was more affected. On the other hand, studies have reported changes not only to waves I or III, but also on the absolute latency of all the waves, in a group exposed to solvents^11^, ^12^. Differently from findings of previous studies, one study reported a statistically significant difference in the increase of the absolute latencies of waves III and V^13^. On the other hand, in a previous study^6^ there were no changes on the absolute latencies of waves, although four individuals had waves with little reproducibility and poor morphology.

Studies with guinea pigs carried out by means of BAEPs showed important results in relation to the neurotoxic action of solvents. In studies with guinea pigs exposed to toluene, there was a worsening in the electrophysiological threshold investigated by means of BAEPs, however, without changes to the absolute and interpeak latencies^14^. One study^15^ assessed adult rats exposed to toluene by means of auditory evoked potentials of the inferior colliculus and histological analysis of the cochlea. Electrophysiological data showed that a concentration of at least 1,500 ppm is necessary to obtain a significant change in the auditory threshold. A significant auditory deficit happened on the frequency range of 8 to 24 KHz. Permanent change in the threshold on the same range of frequency was seen in guinea pigs exposed to toluene and to noise, where the noise alone did not cause changes to the electrophysiological threshold, but damaged the outer hair cells (OHC)^16^. In another study with guinea pigs^17^ there was also change to the threshold of guinea pigs exposed to noise and toluene, and the frequencies of 12.5; 16 and 20 kHz were the most affected ones. According to the authors, the synergic action between noise and toluene became evident in the concentration starting from 1,500 ppm. We assessed rats and chinchillas^18^ by means of the BAEP after exposure to toluene. There was a significant change to the threshold of frequencies 0.5, 1.2, 4, 8 and 16 kHz only in rats. The group of chinchilla did not suffer any auditory effect. The authors suggest that the rat's system is more similar to that of humans, since they eliminate the solvents using the same process.

In relation to interpeak latencies, in the present study we found alterations in all the intervals, in both ears. On the right ear, the I-III interpeak interval had the largest number of subjects with changes (38.1%). On the left ear, the I-III and III-V intervals had the same number of subjects with changes (14.3%). The occurrence of subjects with changes in their interpeak intervals was not statistically significant (*p*>0.05). However, another study^10^ found a statistically significant difference in all the interpeak intervals of rotogravure workers exposed to toluene. In a study involving workers from a printing shop exposed to neurotoxic agents, compared to the control group, they noticed a III-V interpeak latency significantly higher in exposed individuals^11^. Individuals who abuse organic solvents had delays in their I-III and I-V interpeak intervals at 90 dBHL, when compared to the control group^13^.

Considering all the individuals assessed in this study (n = 21), 19% had alterations in wave V interaural difference, although without statistically significant difference (*p*>0.05) (Figure 3). The interaural difference of wave V above 0.3 ms suggests retrocochlear pathology^19^.

Thus, the results from the wave V interaural difference found in the subjects of this study suggest retrocochlear alterations as a consequence of exposure to fuels, even without the synergic interaction of noise and the chemical product, found in most of the current studies.

The auditory thresholds of all the subjects in this study were normal; nonetheless, some had BAEP changes. This finding corroborates those from another study^10^, which also reported central auditory changes in a group of workers exposed to toluene, before the appearance of clinical signs in hearing. The central changes in subjects exposed to solvents may happen, not only on the central auditory system. Confirming such findings using MRI,^20^ there were neurological abnormalities in 50% of the patients assessed (n = 20), which had a past of acute exposure to organic solvents.

The subjects in this study did not have hearing loss, only changes in their interpeak and absolute latencies of waves upon BAEP. However, researchers^8^, ^9^ state to be necessary, respectively, three and five years of exposure to solvents for hearing loss to appear.

Therefore, in regards of exposure duration, the findings from the present study suggest that individuals exposed to solvents for at least three years may have alterations in their auditory nerves (wave I), on the high brainstem (III-V statistically significant - *p* = 0.0257) and alterations on the lateral lemniscus (wave V - *p* = 0.0257). In addition, it has been found that individuals exposed for three years and one month to five years may have alterations in all the structures assessed by BAEP, except the cochlear nucleus. It has also been reported that subjects exposed for more than five years may have diffuse alterations in their brainstem (I-V and III-V interpeaks), besides changes to the peripheral portion of their auditory nerves (wave I). The subjects from this group, may still have changes to the lateral lemniscus, since everyone had changes to the wave V absolute latency.

BAEP findings from the present study suggest the neurotoxic action of organic solvents, which may be identified before clear clinical signs may be noticed, since all the subjects assessed had normal auditory thresholds, nonetheless, with changes in the BAEP results. By the same token, one study^21^ reported normal auditory thresholds in some subjects exposed to solvents, nonetheless, with changes in the digit dichotic test, which also assesses part of the central auditory pathway. Thus, the author suggests that two independent mechanisms may be associated with solvent-induced hearing loss. One mechanism may induce only cochlear dysfunction, while the other only the central auditory dysfunction. Thus, both mechanisms and variables, such as the individual's susceptibility, may be taken into account in order to clarify the differences between the toxicity processes and, thus, identify which is the prevalent mechanism.

## CONCLUSION

Gas station attendants exposed to fuel for a minimum time of three years, even with normal auditory thresholds, may suffer changes in their central auditory system, shown in this study by the increase in absolute latencies, interpeak intervals and interaural difference on the waves assessed by the Brainstem Auditory Evoked Potentials.
